# Genome-wide characterization of genetic diversity and population structure in *Secale*

**DOI:** 10.1186/1471-2229-14-184

**Published:** 2014-08-01

**Authors:** Hanna Bolibok-Brągoszewska, Małgorzata Targońska, Leszek Bolibok, Andrzej Kilian, Monika Rakoczy-Trojanowska

**Affiliations:** 1 Department of Plant Genetics, Breeding and Biotechnology, Faculty of Horticulture, Biotechnology and Landscape Architecture, Warsaw University of Life Sciences, Warsaw, Poland; 2 Department of Silviculture, Faculty of Forestry, Warsaw University of Life Sciences, Warsaw, Poland; 3 Diversity Arrays Technology Pty. Ltd, Yarralumla ACT, Australia

## Abstract

**Background:**

Numerous rye accessions are stored in *ex situ* genebanks worldwide. Little is known about the extent of genetic diversity contained in any of them and its relation to contemporary varieties, since to date rye genetic diversity studies had a very limited scope, analyzing few loci and/ or few accessions. Development of high throughput genotyping methods for rye opened the possibility for genome wide characterizations of large accessions sets. In this study we used 1054 Diversity Array Technology (DArT) markers with defined chromosomal location to characterize genetic diversity and population structure in a collection of 379 rye accessions including wild species, landraces, cultivated materials, historical and contemporary rye varieties.

**Results:**

Average genetic similarity (GS) coefficients and average polymorphic information content (PIC) values varied among chromosomes. Comparison of chromosome specific average GS within and between germplasm sub-groups indicated regions of chromosomes 1R and 4R as being targeted by selection in current breeding programs. Bayesian clustering, principal coordinate analysis and Neighbor Joining clustering demonstrated that source and improvement status contributed significantly to the structure observed in the analyzed set of *Secale* germplasm. We revealed a relatively limited diversity in improved rye accessions, both historical and contemporary, as well as lack of correlation between clustering of improved accessions and geographic origin, suggesting common genetic background of rye accessions from diverse geographic regions and extensive germplasm exchange. Moreover, contemporary varieties were distinct from the remaining accessions.

**Conclusions:**

Our results point to an influence of reproduction methods on the observed diversity patterns and indicate potential of *ex situ* collections for broadening the genetic diversity in rye breeding programs. Obtained data show that DArT markers provide a realistic picture of the genetic diversity and population structure present in the collection of 379 rye accessions and are an effective platform for rye germplasm characterization and association mapping studies.

## Background

Rye (*Secale cereale* L.; 2n = 14, RR) is an out-crossing, wind-pollinated temperate zone cereal with low water and soil fertility requirements and good tolerance for biotic and abiotic stresses. It is an important crop in several Eastern, Central and Northern European countries with cultivation area of approximately 5 Million hectares worldwide in 2011 [http://faostat.fao.org]. The primary uses of rye include bread making, alcohol production, and animal feed. Recently it is also gaining attention as a biomass crop. Rye products are a valuable diet component due to high dietary fiber content and rye bread was shown to have beneficial influence on human health [1,2]. The crop is also of interest to triticale (x *Triticosecale* Wittmack) and wheat (*Triticum* ssp.) geneticists and breeders as a source of genetic variation, since rye is a donor of the R genome of the triticale and the 1RS chromosome is one of the most frequently used sources of alien chromatin in wheat varieties [3,4].

Apart from *Secale cereale* L., three other species are currently recognized in the genus *Secale*: *S. sylvestre* Host, *S. vavilovii* Grossh. (both annual and self-pollinating), and *S. strictum* (C. Presl.) C. Presl. – perennial and open-pollinating. There are eight subspecies in *S. cereale* and five in *S. strictum* with *Secale cereale* ssp. *cereale* L. being the only cultivated rye [5].

Cultivated rye is believed to have originated from south-western Asia from where it was introduced via Russia to Poland, Germany and subsequently distributed throughout most of Europe. It is also hypothesized that there was a second route of migration of the species into Europe – via Turkey and across the Balkan Peninsula [6].

Traditional rye varieties are panmictic populations, characterized by high levels of heterozygosity and heterogeneity [7]. Over 20 years ago hybrid varieties were introduced and quickly gained popularity due to considerable increase in grain yield. Hybrid rye breeding became possible by using an Argentinean landrace as source of a cytoplasmic male sterility [8], while the most effective restorer genes originated from Iranian and South American collections [9].

Early landrace varieties and wild ancestors provide a broad representation of the natural variation that is present in a species and it was demonstrated that they contain genomic segments that can enhance the performance of some of the world’s most productive crop varieties [10]. Increasing the extent of genetic variation available in rye breeding programs by utilization of exotic/primitive accessions is a particularly challenging task because, apart from being agriculturally poor-adapted, the materials in question are also heterozygous and self-incompatible. Nevertheless, it was shown that successful exploitation of these genetic resources is possible in rye even for quantitative traits – promising results were obtained through implementation of marker assisted backcrossing in a study aimed at improving baking quality, where a heterozygous Iranian primitive population was used as a donor [9].

Over eighty rye germplasm collections are maintained world-wide, with the total number of accessions estimated to be approximately 21 000 [11]. Characterization of genetic variation contained in germplasm collections is essential for efficient genebank management [12]. It is also crucial for effective utilization of the genetic resources available in breeding [13]. Over the years several molecular studies were undertaken to asses genetic diversity and relationships in *Secale* species, varieties and inbred lines. Various methods such as, Amplified Fragment Length Polymorphism (AFLP), Inter Simple Sequence Repeat (ISSR), Random Amplified Polymorphic DNA (RAPD), Selective Amplification of Microsatellite Polymorphic Loci (SAMPL), Sequence Specific Amplification Polymorphism (SSAP) and Simple Sequence Repeats (SSR) have been used for this purpose [5,6,14-17]. Analyses of chloroplast an mitochondrial genomes were also done [18]. However, the number of genotypes and the number of marker loci assessed in these studies were usually low. Additionally, in the majority of the studies the markers used were of anonymous nature, with no information on their chromosomal location available. To our knowledge no extensive assessment of genetic diversity represented by any of the *Secale* genebanks was done up to date.

Recently, Diversity Arrays Technology (DArT) markers were developed for rye and their efficacy in detecting genetic diversity in this species was demonstrated [19]. Subsequently a DArT marker based consensus genetic map was constructed [20]. In consequence the chromosomal location of over four thousand DArT markers was determined and genome-wide genomic analyses became available for rye. The DArT method itself was developed in early 2000. It allows for simultaneous detection of several thousand DNA polymorphisms arising from single base changes and indels by utilizing selective hybridization to DNA fragments immobilized on solid-phase slides. Contrary to the other existing SNP genotyping platforms DArT does not rely on sequence information [21]. DArT markers have been successfully used for a variety of genetic studies including construction of high density linkage maps, association mapping and genomic selection [20,22-24]. Recently DArT markers find increasing use in extensive analyses of genetic diversity and population structure of various crops [12,25-29].

The present study was undertaken to analyze genetic diversity and population structure in a set of 379 diverse rye accessions using high density, genome-wide distributed DArT markers. In particular the objectives of the study were: a) to assess the genetic diversity represented by rye accessions from the collection of Polish Academy of Sciences Botanical Garden-Center for Biological Diversity Conservation in Powsin (PAS BG, Warsaw, Poland) including wild, primitive and historic cultivated germplasm from diverse geographic regions, b) to assess the level of genetic diversity represented by the rye varieties currently marketed in Central Europe, also in relation to diversity contained in the germplasm from an *ex situ* collection (c) to compare the distribution of DNA polymorphisms among rye chromosomes.

## Methods

### Plant material

Plant material consisted of 379 rye accessions: 153 landraces, 46 cultivated materials, 137 varieties, 26 breeding strains, and 17 wild accessions. A total of 306 accessions originated from the collection of the PAS BG, the remaining 73 forms were kindly supplied by breeding companies and by Prof A. Lukaszewski (University of California, Riverside). The samples of *Secale cereale ssp. cereale* from PAS BG were selected for maximum diversity and to represent a broad range of historic rye germplasm and geographical regions. Breeding companies supplied rye varieties, which are currently registered and marketed in Europe, including population and hybrid varieties. Detailed information on rye accessions used, including source, improvement status and region of origin is in Additional file 1: Table S1.

### DNA isolation and genotyping

DNA was isolated from ca. 2 weeks old greenhouse grown plants using Mag-Bind Plant DNA 96 kit (Omega Bio-Tek). Each rye accession was represented by 96 plants. DNA isolates were pooled into one representative DNA sample for each accession. DNA samples were genotyped with DArT markers using the procedure and the rye genotyping array described in [20] at Diversity Arrays Technology Pty. Ltd., Yarralumla ACT, Australia (Additional file 1: Table S1). For quality control 33% of DNA samples were genotyped in full technical replication. The reproducibility parameter threshold was set at 95%. Chromosomal locations of DArT markers were obtained from the rye consensus map [20] and the results of analysis of wheat-rye addition lines [19].

### Genetic diversity and population structure

Polymorphic information content (PIC) was calculated for each DArT marker according to Alheit *et al.*[25]. Since DArTs are biallelic dominant markers, PIC values range from 0 (in case of fixation of one allele) to 0.5 (when the frequencies of both alleles are equal). Genetic similarity (GS) matrices based on the Jaccard’s similarity coefficient [30] were constructed in NTSYS-pc 2.1 [31] based on combined information from all polymorphic markers with defined chromosomal location and also separately based on markers from individual chromosomes. Mantel’s test was performed in NTSYS-pc 2.1 to estimate the correlation between the matrices of genetic similarity obtained using marker data from individual chromosomes. The distance (1-Jaccard) matrix based on combined data was used to construct a dendrogram in MEGA 5.1 [32] software with the Neighbor Joining method. Additionally, a dendrogram including only wild *Secale* accessions was constructed to verify their genetic relationships. Two varieties - Petkus from PAS BG and Dankowskie Nowe from Danko were included in this analysis as the representative accessions of *S. cereale* ssp. *cereale*.

The STRUCTURE [33] software was used to identify the number of populations (K) capturing the major structure in data. We used the admixture model, a burn-in period of 10,000 MCMC iterations and 100,000 run length. Five independent runs were performed for each simulated value of K ranging from 1 to 20. The most likely number of K was then determined using the DeltaK method [34] with the help of the Structure Harvester software [35]. Permutations of the output of the STRUCTURE analysis were done with CLUMPP software [36] using independent runs to obtain a consensus matrix. A bar graph of the population structure results was generated with Distruct software [37].

GenAlEx v.6.501 [38,39] was used to assess the amount of variation among the inferred populations by AMOVA and to calculate the pairwise Phi_PT_ values. Phi_PT_, an analogue of F_ST_, is the estimate of population genetic differentiation provided by GenAlEx when binary data are analyzed. GenAlEx was also used to investigate graphically the genetic relationships amongst the rye accessions via principal coordinates analysis (PCoA).

## Results

### Genetic diversity patterns

In total 1054 polymorphic DArT markers with defined chromosomal location were found using the scoring reproducibility threshold of 95%. The average scoring reproducibility was 97.7%. The number of markers per chromosome ranged from 112 for 1R to 231 for 4R. The distribution of markers available by chromosome is given in Table 1.

**Table 1:** Distribution of DArT markers available by chromosome

**Chromosome**	**No. of DArT markers**
1R	112
2R	125
3R	132
4R	231
5R	122
6R	204
7R	128
total	1054

**Figure 1:**
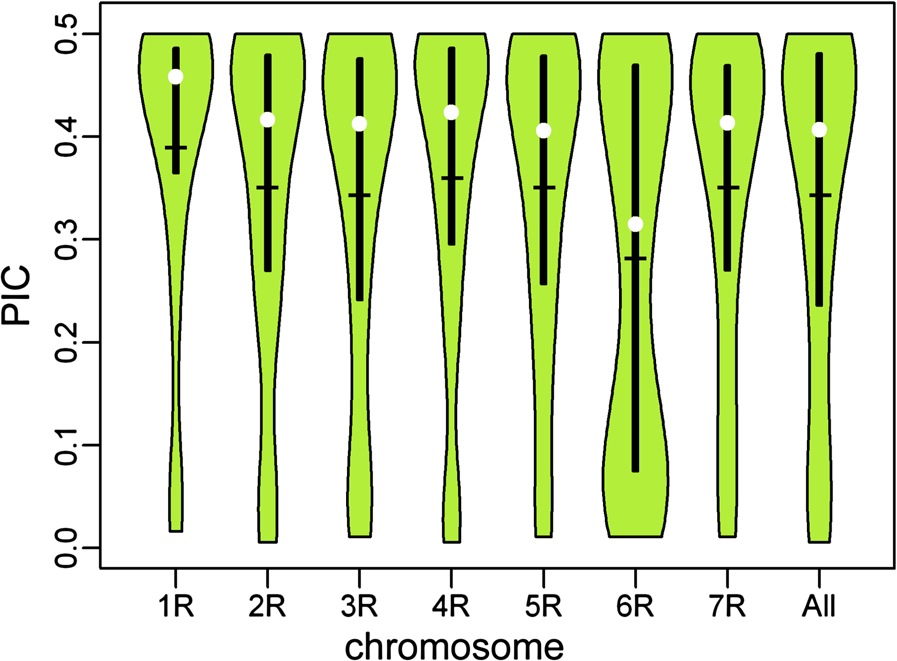
**Distribution of PIC values**, **by chromosome and for all markers.** Violin plots show density distribution of PIC values, horizontal bar indicates average value, median is shown as white circle, top and bottom of vertical bar represent the first and third quartile.

The mean PIC value for all markers with chromosome location was 0.34. PIC values observed in our study ranged from 0.01 to 0.50 with a high proportion o markers with PIC values above 0.41 (51.7%). PIC values varied among chromosomes, from 0.28 for 6R to 0.39 for 1R. Markers from individual chromosomes exhibited similar PIC values distributions with exception of chromosome 6R, where a large proportion of markers with PIC value below 0.1 (ca. 29%) was observed. Chromosome 1R was characterized by the highest proportion of markers with PIC values above 0.41 (ca. 68%) and the lowest proportion of markers with intermediate PIC values. Violin plots showing distribution of PIC values by chromosome and chromosome specific averages are shown in Figure 1. Mean PIC values for all markers with chromosome location were also calculated separately for sub-groups of accessions created according to source and type of germplasm and varied from 0.26 for varieties from breeding companies to 0.39 for wild accessions. (Additional file 2: Table S2).

**Figure 2:**
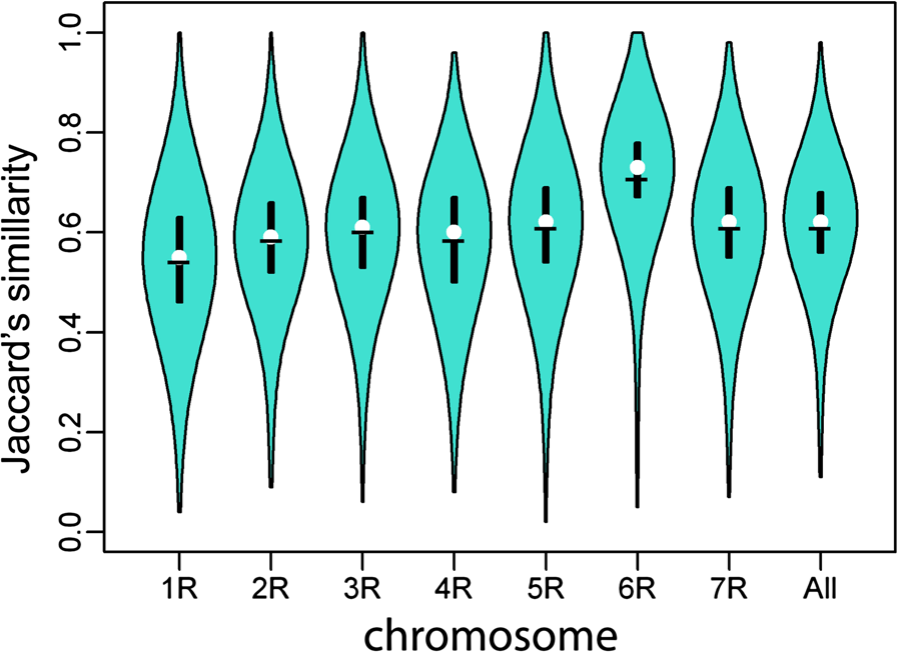
**Distribution of GS values**, **for all markers and by chromosome.** Violin plots show density distribution of GS values, horizontal bar indicates average value, median is shown as white circle, top and bottom of vertical bar represent the first and third quartile.

**Figure 3:**
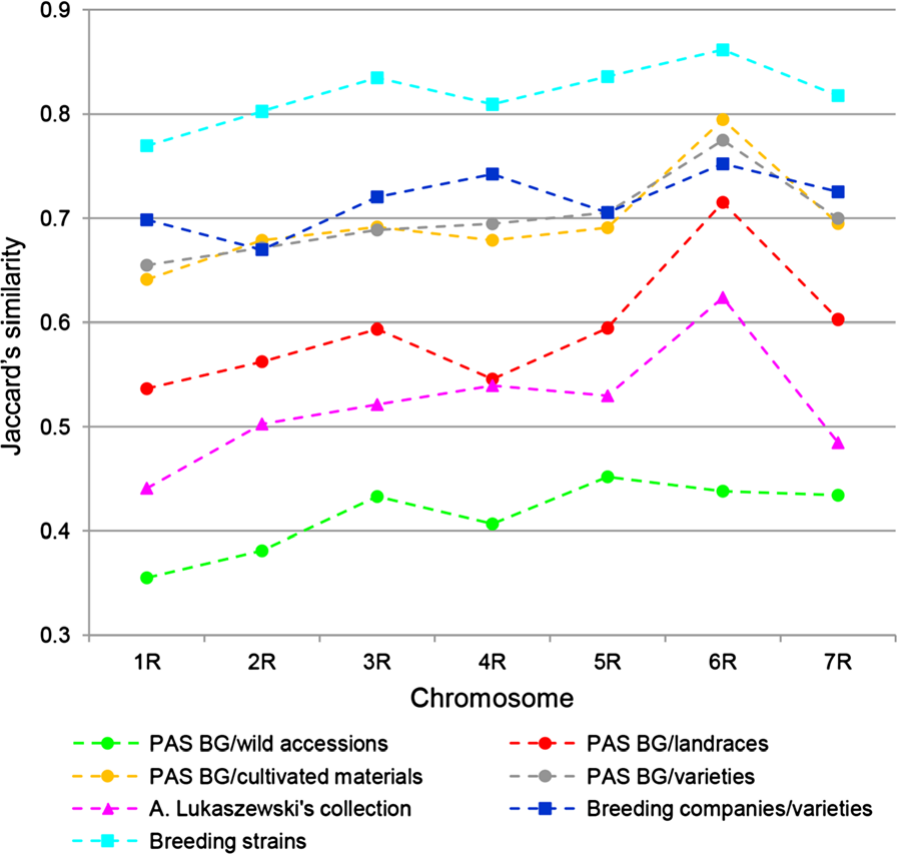
Average GS values for groups of accession by chromosome.

GS values calculated based on all markers varied from 0.11 to 0.98 with the average 0.61. In the case of the individual chromosomes the highest average GS (0.71) was observed for chromosome 6R, the lowest (0.54) for chromosome 1R. While the combined markers were able to differentiate all accessions, markers in chromosome specific sets (with exception of markers from 4R and 7R) were unable to distinguish two accessions of *S. sylvestre* included in the study. Noteworthy is the relatively low GS value for two samples of Dankowskie Nowe variety, one from PAS BG and the other one from the breeding company Danko, equal 0.82, obtained using combined marker data. The average GS values for individual chromosomes were significantly different (p = 0.01), except for the average GS for chromosomes 5R and 7R. Violin plots showing distribution of GS values by chromosome and chromosome specific averages are shown in Figure 2. The average GS values were also calculated for 7 sub-groups of accessions created according to the source and type of germplasm (Figure 3). The pattern of differences in chromosome specific average GS was moderately consistent in different germplasm sub-groups. The highest average GS values were observed for chromosome 6R for all sub-groups, with exception of wild accessions, where the highest GS value was obtained for 5R. The lowest average GS for accession groups occurred in the case of chromosome 1R, except for varieties supplied by breeding companies. In this sub-group the lowest average GS was recorded for chromosome 2R. Overall the lowest average GS was observed for wild accessions, followed by accessions from A. Lukaszewski’s collection. The highest average GS was obtained for breeding strains from Danko. Compared to the pattern of chromosome specific average GS values observed in germplasm sub-groups a markedly higher average GS value was observed in 4R for varieties from breeding companies. In general, the average GSs values for varieties and cultivated materials from PAS BG and for varieties from breeding companies were similar, especially in the case of chromosomes 2R and 5R.

The results of the Mantel’s test for correlation between the matrices of genetic similarity obtained for individual chromosomes were significant (p = 0.001) and positive with the correlation coefficient r values ranging from 0.64 for 1R and 7R to 0.77 for 2R and 6R (Table 2).

**Table 2:** Correlations of GS matrices obtained with chromosome specific marker sets

	**1R**	**2R**	**3R**	**4R**	**5R**	**6R**
2R	0.71					
3R	0.72	0.75				
4R	0.72	0.75	0.72			
5R	0.66	0.72	0.72	0.69		
6R	0.68	0.77	0.75	0.75	0.71	
7R	0.64	0.71	0.72	0.67	0.72	0.73

### Model-based population structure

**Figure 4:**
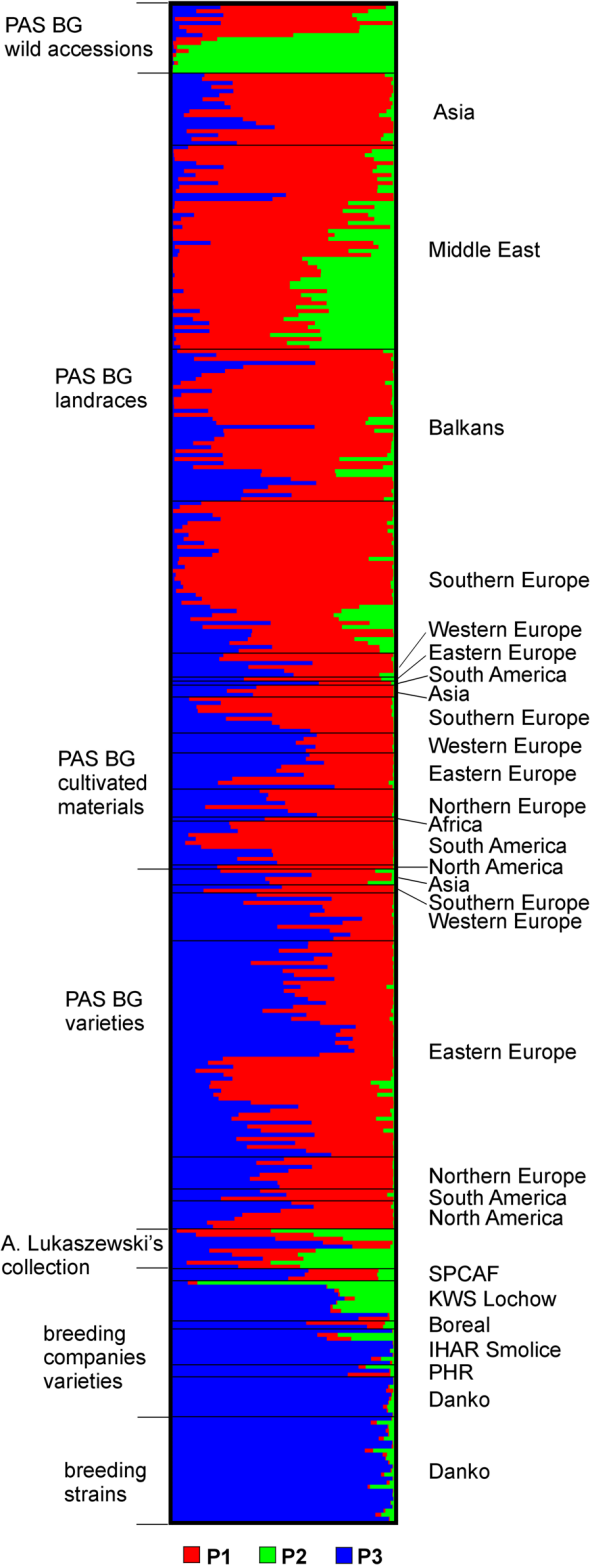
**STRUCTURE plot of the 379 rye accessions with K = 3 clusters based on 1054 DArT markers.** The plot is sorted according to accession source and type. The order of accessions in the plot corresponds to the order of accessions in Additional file 1: Table S1. Each accession’s genome is represented by a single row, which is partitioned into colored segments in proportion to the estimated membership in the three subpopulations. Black line separates accessions of predefined groups.

It was estimated through the method of Evanno *et al.*[34] that there are 3 groups contributing significant genetic information in the analyzed *Secale* collection (Additional file 3: Figure S1). STRUCTURE results, grouped and graphed according to accession source, type and geographic region are shown in Figure 4. The classification of accessions into populations by the model-based method is given in Additional file 1: Table S1. In total 226 accessions (59.6%) were assigned to one of the three populations, where at least 70% of their inferred ancestry was derived from one of the three model-based populations. Populations 1, 2, and 3 (P1, P2, and P3) consisted of 140, 10 and 76 accessions, respectively. The remaining accessions were categorized as having admixed ancestry, including 33 admixtures between P1 and P2 (P1P2), 114 between P1 and P3 (P1P3), and two between P2 and P3 (P2P3). Four accessions had similar percentages of their inferred ancestry derived from each of the three model based population and were classified as heterogeneous (H).

Population assignments of accessions from different germplasm sub-groups were as follows: wild *S. cereale* accessions (with exception of *S. cereale* ssp. *ancestrale*, categorized as heterogeneous) and *S. vavilovii* accessions were assigned to P1, whereas accessions of *S. strictum* and S. *sylvestre* were assigned to P2. Landraces were mostly categorized as P1 (ca. 60% landraces from different regions) The remaining landraces were classified as P1P3 (ca. 18% landraces from various regions) and P1P2. (also ca. 18%). Landraces constituted the majority of the later subpopulation (ca. 85%), and most of them (23 accessions – ca. 66% of all Turkish landraces in the collection) originated from Turkey. Cultivated materials had mostly admixed ancestry – almost 59% of accessions from this sub-group were classified as P1P3, the rest was assigned to P1. Similarly, the majority of varieties from PAS BG (61%) were assigned to P1P3. About 20% of varieties from PAS BG was categorized as P1, and ca. 19% – as P3. The assignment of cultivated materials and varieties from PAS BG to different subpopulations appeared to be rather uncorrelated with the geographical origin of accessions. All breeding strains and the vast majority of the varieties from breeding companies (84%) were categorized as P3. All three varieties from Belarus and one of the varieties from Boreal – Rihii – were classified as admixtures P1P3. Only one variety from breeding companies –KWS Magnifico F_1_ from KWS Lochow was classified as admixture P2P3. Noteworthy is that F_1_ varieties included in the set and assigned to P3 had a relatively high proportions of alleles from P2 – from 18 to 30% (Figure 4). The population varieties from breeding companies, that were assigned to P3, had the percentage of their inferred ancestry derived from that population close to 100%. A single variety – Gonello F_1_ from KWS Lochow – was assigned to P2. Accessions from A. Lukaszewski’s collections had mostly admixed ancestry and myriad subpopulation assignments, with three accessions classified as heterogeneous. In general the classification of populations appeared rather uncorrelated with the geographical origin of rye accessions, but rather reflected the source of the accessions, and, to a degree, the improvement status of the accessions.

### Relationships among accessions based on PCoA and cluster analysis

**Figure 5:**
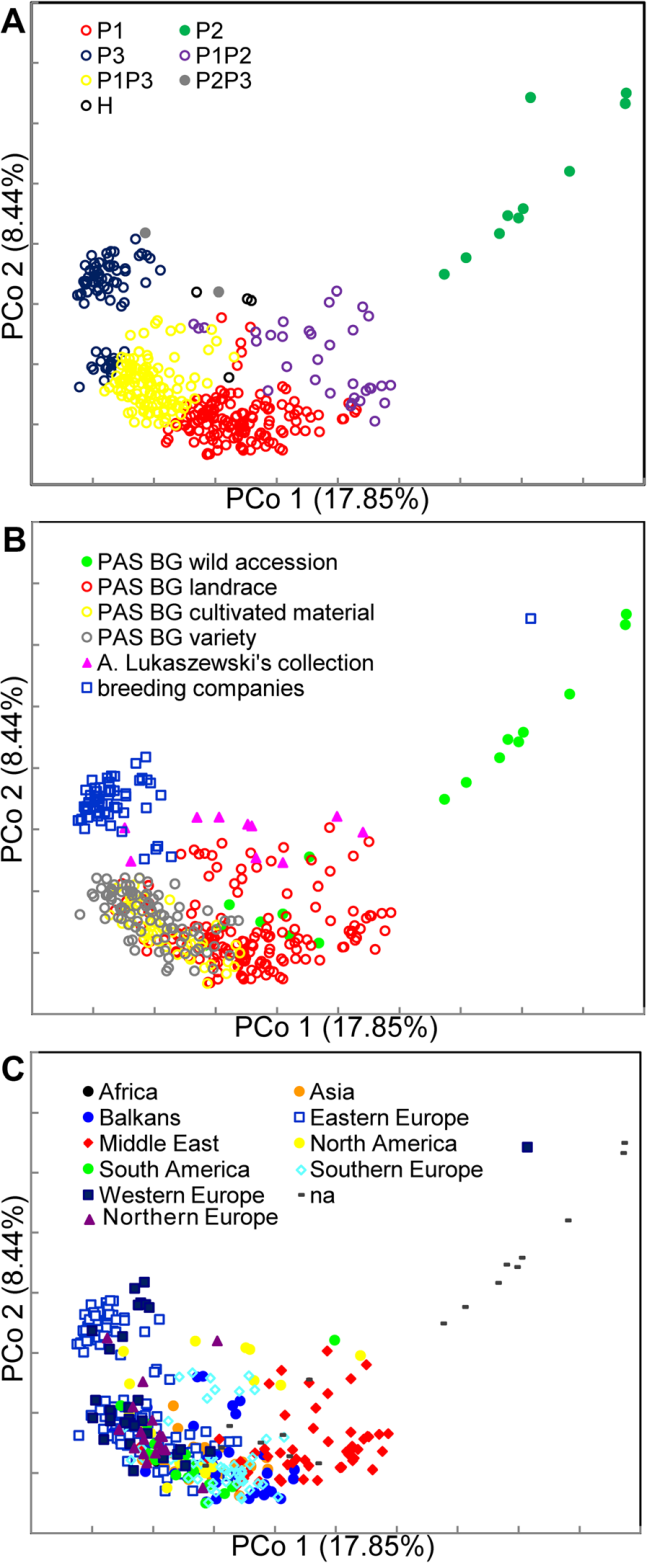
**Principal coordinate analysis of 379 rye accessions based on 1054 DArT markers.** Panel **A**: accessions were labeled according to the STUCTURE results; panel **B**: accessions were labeled according to the source and improvement status; panel **C**: accessions were labeled according to the geographic origin.

The PCoA was largely consistent with the STRUCTURE results (Figure 5A). The percentages of genetic diversity explained by the first and the second coordinate were 17.85 and 8.44, respectively. The three model-based populations were well separated with admixtures and heterogeneous accessions located between populations. However, accessions classified as P3 based on STRUCTURE results were divided into two groups in the PCoA plot. One of the resulting groups contained all the materials from breeding companies assigned to P3, and was separated from all remaining accessions from the study. The second group consisted of all the varieties from PAS BG that were assigned to P3 and was located adjacent to the group of accessions from PAS BG classified as admixtures P1P3.

We also used PCoA to determine the extent to which the accessions from different sources and of different improvement status represent distinct areas of diversity space (Figure 5B). It was revealed that, indeed, most materials supplied by breeding companies (which included also several older, but still marketed varieties, such as Dankowskie Nowe and Amilo - release year 1976 and 1987, respectively), occupied a distinct area of diversity space, well distinguished from the remaining accessions. Varieties and cultivated materials from PAS BG overlapped in the PCoA plot, and occupied a relatively narrow space. Landraces represented much greater diversity, with some overlapping with PAS BG varieties and cultivated materials and with accessions from A. Lukaszewski’s collection, which in turn occupied a space between materials from other sub-groups. Accessions of *S. sylvestre*, and *S. strictum* were separated from the rest of the rye accessions, whereas wild *S. cereale* and *S. vavilovii* accessions, were intermixed with landraces. Thus, it can be concluded that source and improvement status contributed significantly to the structure observed in the analyzed set of *Secale* germplasm.

**Figure 6:**
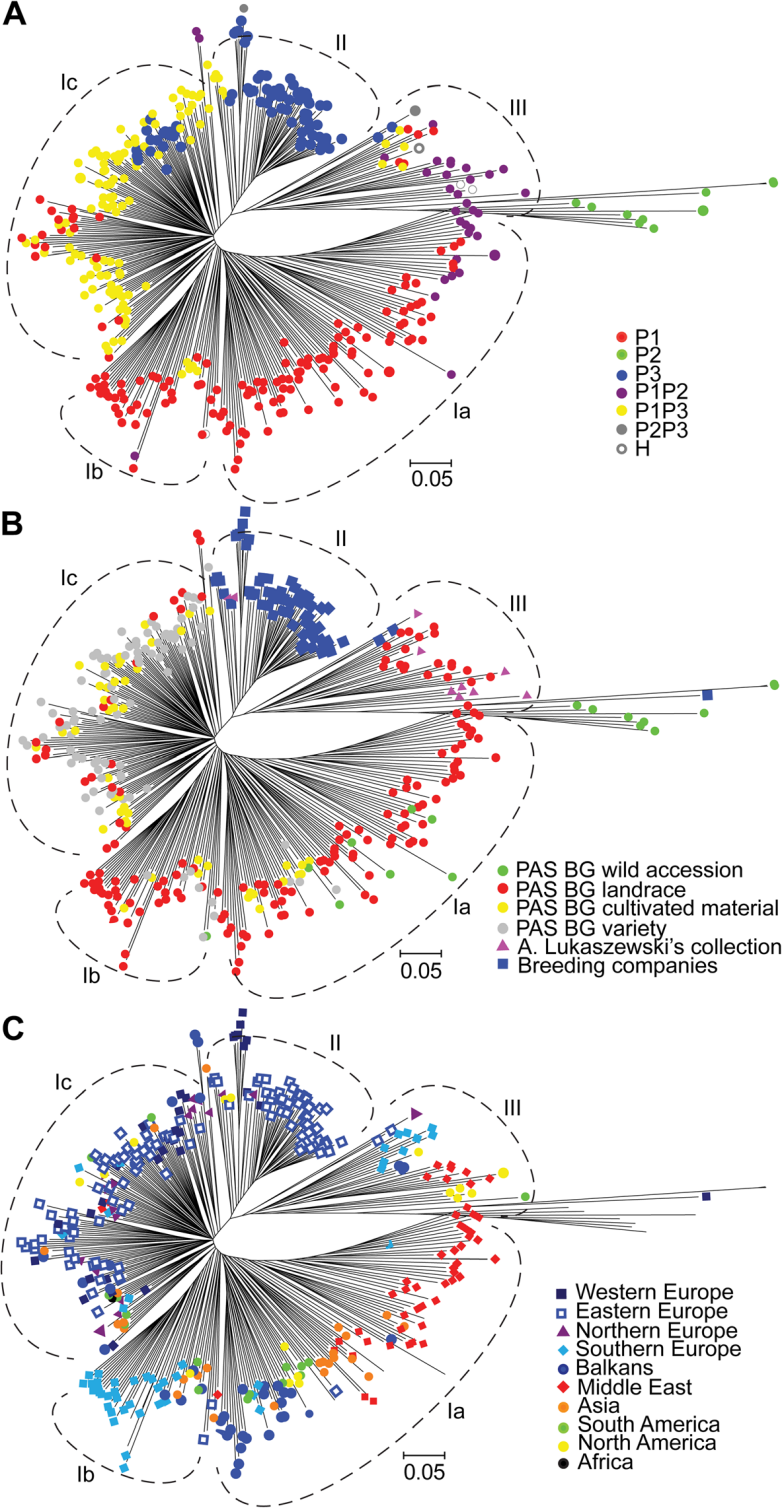
**Dendrogram demonstrating the genetic relationships among 379 rye accessions based on 1054 DArT markers**, **obtained using Neighbor Joining clustering from Jaccard**’**s dissimilarity matrix.** Panel **A**: accessions were labeled according to the STUCTURE results; panel **B**: accessions were labeled according to the source and improvement status; panel **C**: accessions were labeled according to the geographic origin.

On the other hand the distribution of the rye accessions in the 2D space appeared largely unrelated to geography (Figure 5C). It is particularly evident in varieties and cultivated materials from PAS BG, with accessions of different origins dispersed and intermixed in the space occupied by these sub-groups (Additional file 4: Figure S2a). Nevertheless in the case of landraces a certain separation between European and Middle Eastern accessions can be observed. Additionally, Asian landraces clustered relatively close together and occupied an area, where the European and Middle Eastern clusters overlapped (Additional file 4: Figure S2b). Based on PCoA landraces from South Europe, Balkans and Middle East were the most diverse subset of *S cereale* ssp. *cereale* accessions from PAS BG analyzed in this study, with a subset of Middle Eastern landraces (corresponding to the sub-group P1P2 inferred by STRUCTURE analysis) representing a distinct diversity space, not overlapping with other accessions.Clustering analysis based on Neighbor Joining allowed the detection of three major clusters: I, II and III (Figure 6). Cluster I, containing the majority of the accessions, could be further subdivided into three clusters a, b and c. The clustering of accessions in the unrooted Neighbour Joining tree was generally in agreement with the model-based population structure of the collection (Figure 6A). The inferred sub-populations were relatively well but not completely separated. Accessions from sub-population P1 were located mostly in clusters Ia and Ib, P2 in cluster III, P3 mostly in cluster II with a subset of accessions in cluster Ic. This separation of P3 into two sub-groups was consistent with PCoA results. Admixtures P1P3 were located mostly in cluster Ic, while the admixtures P1P2 grouped on the verge of cluster Ia, and also in cluster III adjacent to P2 accessions.The Neighbour Joining tree topology reflected the source and the improvement status of the accessions (Figure 6B), with the accessions from breeding companies placed almost exclusively in cluster II, the varieties from PAS BG mostly in cluster Ic and the accessions from A. Lukaszewski’s collection in cluster III. Cultivated materials were dispersed in clusters Ia, Ib and Ic. Landraces constituted the majority of the accessions in clusters Ia and Ib, although several landraces occurred also in clusters Ic and III.Correlation of clustering with geographic region of origin was rather week (Figure 6C). It was slightly more pronounced than in the case of PCoA, but also mostly restricted to landraces. A separation of Middle Eastern and Balkan accessions was visible in cluster Ia. Cluster Ib contained mostly Southern European accessions.

**Figure 7:**
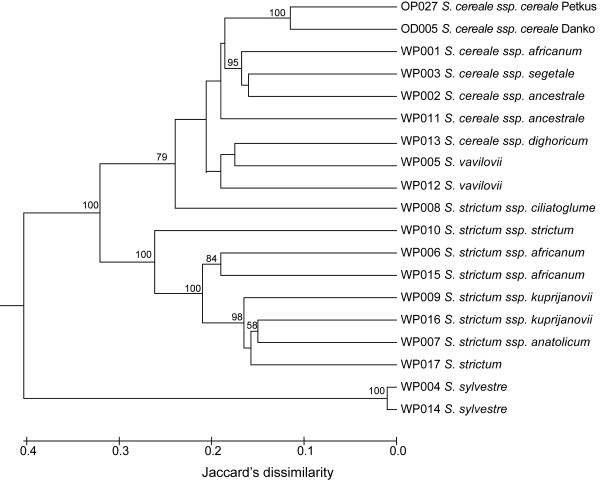
**Dendrogram demonstrating genetic relationships among wild rye accessions based on Jaccard**’**s dissimilarity matrix.** Bootstrap support values are shown if greater than 50%.

The DArT markers based hierarchical clustering of wild *Secale* accessions (Figure 7) revealed that *S. sylvestre* samples were very divergent from the rest and formed a separate group. The remaining accessions formed two clusters. One of them comprised only *S. strictum* accessions, the second cluster consisted of *S. cereale* subspecies, both *S. vavilovii* samples and *S. strictum ssp. ciliatoglume*.

### Genetic differentiation among accession sub-groups

AMOVA analysis of the model-based populations P1, P2 and P3 showed that there was a much greater proportion of variation within populations (75%) than among populations (25%, P < 0.001). Pairwise Phi_PT_ values indicated a high degree of differentiation between the populations P1 and P3 (0. 21) and a very high genetic differentiation between population P2 and populations P1 and P3 (0.36 and 0.53, respectively, P < 0.001). AMOVA analyses were also done to assess genetic differentiation among accessions grouped according to geographic region of origin and germplasm source, and improvement status. Again, a much greater proportion of variation within population than among populations was found: 90% and 10%, respectively, (P < 0.001), when accessions were grouped based on region of origin, and 87% and 13%, (P < 0.001), when source and improvement status of accessions were used as criterion for grouping. Based on pairwise Phi_PT_ values, there was a substantial differentiation between wild accessions and the remaining accession sub-groups (Additional file 5: Table S3). Within cultivated ryes high genetic differentiation was observed between accessions from breeding companies and remaining four germplasm groups: landraces, cultivated materials, varieties from PAS BG and accessions from A. Lukaszewski’s collection (Phi_PT_ values ranging from 0.15 to 0.20). By contrast little genetic differentiation (Phi_PT_ values below 0.05) was detected between landraces and cultivated materials, and between cultivated materials and varieties from PAS BG. When the accessions were grouped according to region of origin, the highest pairwise Phi_PT_ values indicating high genetic differentiation were found between accessions from Middle East and accessions from tree European regions: Eastern Europe, Western Europe and Northern Europe (0.21, 0.18 and 0.16, respectively). Little or moderate genetic variation was found between accessions from remaining regions (Additional file 5: Table S3).

## Discussion

DNA markers rapidly became an indispensable tool of assessing genetic diversity contained in germplasm collections that supplements morphological evaluations. While in the early studies the laborious and time consuming procedures of detecting DNA variation allowed for sampling of only relatively limited numbers of accessions and loci, the development of high throughput genotyping methods, (fluorescence-based SSR detection on automated sequencers, and, in particular, highly parallel SNP genotyping assays) enabled a thorough characterization of whole germplasm collections [12,13,25,27,40-44].

The development of high throughput genotyping methods in rye has lagged behind that of other cereals and so far no large scale genetic diversity studies of *Secale* were conducted. The development of DArT genotyping array for rye opened the possibility for genome-wide genetic analyses in this crop [19]. In this study we applied 1054 genome-wide distributed DArT markers with defined chromosome location to assess genetic diversity and population structure in a collection of 379 rye accessions. To our best knowledge it is the most comprehensive study of genetic diversity in rye done until now.

In our study we analyzed bulked DNA samples, originating from 96 plants pro accession. This strategy allows to capture in one sample the genetic variability of a heterogenous accession, which will be of advantage in future analyses of the assembled collection involving multiallelic markers. At the same time, however, this strategy equilibrates a part of genetic diversity of an accession, when, like in our study, dominant markers are used for genotyping. Nevertheless DArT markers performed well in providing the picture of genetic diversity in a large collection of rye germplasm. The combined data allowed us to distinguish all accessions. The average PIC (0.34) was found to be intermediate to that observed in studies of genetic diversity done using DArT markers in other species, such as cassava (0.42) [45], common wheat (0.40) [26], triticale (0.40) [25], barley (0.38) [46], *T. monococcum* (0.31) [28], *Lesquerella* (0.21) [12], sugar beet (0.28) [47] and *Asplenium* (0.21) [48]. The average PIC of rye DArT markers was thus lower than in autogamous crops, whereas open-pollinating species are generally expected to exhibit a higher level of polymorphism that self-pollinating ones [49]. This value was also slightly lower than mean PIC for the R genome (0.38) reported for DArT markers in triticale by Alheit *et al.*[25]. This lower than expected mean PIC value can result from certain limitations of DArT markers in analyses of heterozygous and heterogeneous samples [19], that will be discussed in more detail later, and from germplasm choice [45]. Whereas the accessions chosen for this study were selected to represent maximum diversity, most of them were not previously subjected to extensive molecular or morphometric analyses. Hence the choice of accessions was made mostly based on the available pedigree information and region of origin. Genotyping with genome-wide DArT markers revealed a limited diversity in certain germplasm subgroups, such as breeding strains. This lower diversity, manifested by prevalence of one marker score (1 or 0) in the respective sub-groups, contributed to lower overall PIC values for a subset of DArT markers and, in effect, to a lower mean PIC value. By contrast, mean PIC value calculated based only on markers scores obtained in landraces, the most diverse germplasm subgroup of *S. cereale* ssp. *cereale* analyzed in our study, was higher (0.38). Similarly, in cassava (which is also an allogamous and highly heterozygous crop), a high mean PIC value of 0.42 was obtained when 35% of analyzed samples were wild relatives of cassava, while an approximately 27% lower mean PIC value (0.27) was achieved in an experiment, where wild relatives constituted only 7.9% of the accessions [45]. On the other hand, as noted by Badea *et al.*[50], the lower PIC values of DArT markers lead to a more defined genetic structure of accessions from distant geographic regions or genetic origin and thus can be of advantage in cluster analyses.

The range and distribution of PIC values was similar to those observed in other studies [28,45]. However, in general, a relatively higher proportion of markers with PIC values above 0,4 was observed. Average PIC values varied between chromosomes. Considerable differences in chromosome specific average PIC values were also reported by Alheit *et al*., who performed genome-wide evaluation of genetic diversity in triticale using DArT markers [25]. Similarly to our results the highest average PIC occurred in winter triticale for 1R, but the lowest chromosome specific average PIC value, was observed for 3R. However, due to limited number of rye founder lines used for the establishment of triticale its R genome may not reflect accurately the genetic variation of the rye genome.

The GS values obtained in our study showed a greater range and a lower average than in previous studies on genetic diversity in rye [5,6], which was probably caused by inclusion of diverse wild and primitive accessions in the collection analyzed in our study. Ma *et al*. [6] assessed genetic diversity in 42 spring and winter rye varieties using RAPD markers. The obtained GS values ranged from 0.435 to 0.964, the average GS value was not reported. Shang *et al*. [5] analyzed separately 30 wild *Secale* accessions and 47 cultivated ryes with 24 SSR markers. GS values obtained in their study ranged from 0.326 to 0.932 (0.633 on average) and from 0.622 to 0.921 (0.773 on average) in wild and cultivated accessions, respectively. This difference in average GS between wild and cultivated *Secale* accessions (with the lower average GS in wild ryes) is in agreement with our results on average GS values in different germplasm subgroups. Large scale population genomic analyses have the potential to provide insight into evolution of crop plants and their genomes, since the genomic regions that have been targeted by selection during crop evolution are expected to exhibit characteristic changes in levels of polymorphism [41]. The high average GS value on 6R that occurred in all sub-groups of cultivated germplasm, but was not observed in wild accessions might thus indicate that this chromosome contains regions that were subjected to strong selection pressure during domestication. Similarly, in the case of varieties supplied by breeding companies, the relatively high GS averages on 1R and 4R, that deviated from the general pattern of differences in chromosome specific average GS values observed within germplasm groups might reflect the presence of genomic regions with limited polymorphism, possibly resulting from selection for QTLs located therein [25] and controlling adaptive traits and quality characters relevant for cultivation in Central and Northern Europe. Chromosome 1R is known to contain genes controlling resistance to diseases and insects, improving adaptation and increasing yield [51], while chromosome 4R was found to harbour QTLs for alpha-amylase activity, preharvest sprouting, kernel thickness, heading time, chlorophyll content in leaves, and flag leaf length [52]. The relatively high GS averages on 1R and 4R that occurred in varieties supplied by breeding companies could also result from the locations of self fertility mutations and fertility restoration genes deployed in hybrid rye breeding. According to Lundqvist self incompatibility in rye is controlled by two multiallelic loci – *S* and *Z*. and mutations at these loci lead to self fertility [53,54]. The two loci *S* and *Z* were subsequently mapped on chromosomes 1R and 2R, respectively, and additional self fertility genes on chromosomes 4R, 5R and 6R were also described [55]. It can by hypothesized that self-fertility genes that are captured in commercial hybrid rye breeding programs and allow for development of inbred parental lines are located in chromosomes 1R and/or 4R. Effective fertility restorer genes, originating from Iranian and Argentinean germplasm and currently used in hybrid rye breeding, are located on chromosome 4RL [56,57].

In this work we used simultaneously three methods - Bayesian clustering, PCoA and Neighbor Joining clustering to obtain a picture of genetic relationship in the collection of 379 rye accessions. Despite minor differences, the results were largely consistent. First of all we found that genetic diversity within germplasm sub-groups consisting of improved rye accessions - obtained from breeding companies, as well as historic varieties and cultivated materials from PAS BG - is relatively narrow. It is possible that the genotyping of bulked DNA samples with dominant DArT markers resulted in lower estimates of genetic diversity and further cost- and labor-intensive analyses based on single plants within accessions with a method, which enables detection of heterozygosity would be needed to unequivocally address the question of the relatively high genetic similarity of rye varieties. It is, however, noteworthy that similar results regarding rye varieties were obtained earlier using RAPD markers [6,58], AFLP markers [17], SSR markers [5], in analyses of organellar genome diversity [18] and in a preliminary small scale study on utility of DArT markers for assessment of genetic diversity in rye [19]. It was postulated that this relatively low genetic diversity could indicate common genetic background of improved rye accessions from diverse regions and can be attributed to an extensive germplasm exchange [6,18]. Interestingly, recently this supposition was partially confirmed by SSR analyses of single S_0_ plants form five Eastern European open-pollinated varieties. A close relationship between varieties was revealed, pointing to common ancestors and/or gene flow between the varieties [59]. The relatively high similarity of rye varieties observed in our study, and hypothesized to be indicative of common genetic background and germplasm exchange, is also in agreement with the available information on the rye breeding history and the ancestry of rye varieties. One of the leading rye varieties in the twentieth century was Petkus and many of the open pollinated rye varieties worldwide are selections from Petkus or include Petkus in their ancestry [59-61]. Eastern European population varieties were crossed with each other in the past, and Petkus has a large contribution in all of them [59], while in Germany, before the establishment of hybrid rye breeding, all leading population varieties belonged either to the Petkus or to the Carsten genepool [56]. Moreover introgressions of foreign material were common in Eastern European breeding programs [62].

On the other hand accessions from breeding companies turned out to be distinct from the remaining accessions, both primitive and improved. This could reflect the adaptation to local conditions of Central and Northern Europe and to requirements of modern agriculture. However, in our opinion, it is at least partly attributable to different reproduction methods and to the accuracy of the procedures used by breeders and during genebank maintenance. We are inclined to this supposition based on the results obtained for the two samples of Dankowskie Nowe variety originating from different sources - the relatively low GS value and assignment to different clusters in PCoA and Neighbor Joining analysis. While accessions reproduced by breeders are subjected to conservative breeding, the reproduction of rye accessions in genebanks is carried out on 1 m^2^ field plots and before flowering the whole plot area is covered with a 2-m-high metal frame covered with pollen-proof tissue [63]. Since in our study only one variety was represented by two independent samples of different origin no definitive conclusions can be drawn and additional comparative studies are needed. Nevertheless, changes in allele frequencies after reproduction cycles were discovered by Chebotar *et al*. [7] in rye accessions from the IPK Gatersleben genebank. They also stressed the importance of making extended efforts in order to sustain the genetic identity of open pollinating rye accessions during *ex situ* maintenance, such as using plots large enough for growing a number of plants sufficient to cover the whole diversity of the populations, omitting harvest when a large proportion of plants is lost during a regeneration cycle and dividing the resources into base and active collections.

Another finding concerning accessions supplied by breeding companies is a relatively high proportion of the inferred ancestry derived from P2, that occurred in hybrid varieties. The unique features of hybrid varieties, differentiating them form open pollinated rye varieties and originating from exotic accessions are cytoplasmic male sterility and fertility restoration genes. In our study we used only DArT markers with defined chromosomal location. Therefore only nuclear DNA was analyzed. Fertility restoration genes can account for some proportion of P2 alleles, however, since after only four rounds of backcrossing, the percentage of the donor genome is reduced to 3.125%, it is unlikely that restorer genes alone account for the whole proportion of P2 alleles observed in hybrid varieties. Hence, further analyses would be required to identify the cause of the observed allelic composition.

Another interesting issue is the relation of the obtained results to the available information on the genetic background of hybrid rye varieties. In Germany, based on diallel variety crosses analyzed by Hepting [61], Petkus and Carsten gene pools were chosen for the development of seed- and pollinator lines, respectively, for hybrid rye breeding [56]. In our study, based on STRUCTURE analysis, Petkus together with hybrid varieties, was assigned to population P3, and Carsten’s Roggen was classified as P1P3. While this results indicate close relationship of hybrid varieties to Petkus and Carsten, they do not mirror the expected high genetic divergence between these two varieties, which causes high hybrid performance. Moreover, the Kustro variety, which is a selection from Petkus, contrary to expectations, was not assigned to population P3 alongside Petkus, but, similarly to Carsten, was classified as admixture P1P3. These results should be, however, treated with a certain caution, since, as mentioned before, allelic compositions of these accessions could have changed during gene bank maintenance, and thus can be not equivalent to allelic composition of Carsten and Petkus populations maintained and exploited by breeders. On the other hand our results seem to be in agreement with the information concerning the existence of Easter European gene pools, which proved to be heterotic counterparts to both Petkus and Carsten genepools [56], since several of Eastern European varieties analyzed in our study were assigned to population P1.

A very striking result was obtained for the Gonello F_1_ variety. This variety is advertised as having the highest frost tolerance. Thus it could be hypothesized that its divergence from other currently registered varieties included in our study is a result of using a very distinct genepool as a source of cold resistance genes. Unfortunately such supposition is not supported by the available information on the genetic background of the hybrid varieties from KWS Lochow: Gonello F_1_ ((Lo115-P × Lo133-N) × LSR83 (Lo310 × Lo312)) is closely related to Guttino F_1_ ((Lo115-P × Lo133-N) × LSR81 (Lo298 × Lo312)), and the male line Lo310 is included in the pedigree of Palazzo F_1_. Both Guttino F_1_ and Palazzo F_1_ were analyzed in our study and clustered closely with other varieties from breeding companies. Hence, additional studies involving Gonello F_1_ are needed to verify the obtained results and to resolve this issue.

Our research showed also that rye landraces represented fairly large diversity. This result is consistent with expectations [10] and also in agreement with the result of a small scale (12 rye accessions) study done using RAPD markers [58]. The fact that the landraces were distant from material obtained from breeders and a subset of landraces occupied a distinct diversity space, not overlapping with other accessions (as shown in PCoA) demonstrates that there is a great genetic potential for detection of unexplored alleles to broaden the genetic diversity in current breeding programs. Distinctness of landraces from contemporary varieties was also observed in tomato [64].

AMOVA results obtained in our study indicate that there is a higher amount of genetic diversity within populations than among population, which is in agreement both with expectations for an out-crossing species [56], and with previous results obtained in genetic diversity analyses in rye using both codominant isoenzyme markers [60], and dominant RAPD markers [58].

In the case of wild *Secale* accessions, the outcome of cluster analysis separating the *Secale* species into three groups – *sylvestre*, *strictum*, and *cereale*/*vavilovii* is in agreement with the results of AFLP and SSR based analyses of phylogenetic relationships in the genus. It is also consistent with the revision of taxonomical classification of *Secale* done by Frederiksen and Petersen [65], who recognized only tree species within the genus *Secale*: *S. sylvestre*, *S. strictum* and *S. cereale*, and included *S. vavilovii* in *S. cereale*. Similarly, Kobyljanskij [66] classified *S. vavilovii* as a subspecies of *S. cereale*. Furthermore, we observed that wild accessions clustered together at almost the same similarity level (about 40%) like in a previous SSR–based study of phylogenetic relationships in the genus *Secale*. It might suggest that the set of biallelic DArT markers used here has a similar discriminatory power as multiallelic SSRs. The fact that *S. strictum* spp. *ciliatoglume* did not cluster with other *strictum* accessions is consistent with the AFLP based results of Chikmawati *et al*. [14], with results of SSCP and SSR analyses done in our laboratory (Targonska *et al*. in preparation), and with the observations of Frederiksen and Petersen, who based on morphometrical analyses suggested that *S. ciliatoglume* should be given an intraspecific rank [67].

Overall we observed rather poor correlation between clustering of rye accessions and geographic origin and lack thereof in rye varieties and cultivated materials. Similar results were obtained earlier in rye using organellar genome analyses [18], SSR markers [5] and AFLP markers [17] and can be explained by common genetic background of the accessions in question resulting from germplasm exchange. Conversely, Ma *et al*. [6] found a good correspondence of clustering to geographical location while analyzing 40 rye varieties mostly of European, North American and Chinese origin. However, they observed that temporal isolation has influenced the genetic diversity of rye more than geographical isolation, since winter and spring varieties formed separate clusters. The growth habit was found to be the primary determinant of population structure also in triticale [25] and barley [68] This issue is however beyond the scope of our study, since the vast majority of the accession were winter types. Our results indicate that the population structure of the assembled rye collection was mostly determined by the accessions source and improvement status and that the significant influence of accessions source seems to be at least partly attributable to the influence of reproduction methods on the genetic identity of rye accessions. This is an interesting and rather unexpected finding and it would be worth wile to verify it in a follow up study, involving the same set of markers and comparative analyses of fairly numerous accessions sets from several rye genebanks, as well as modern rye varieties bred for cultivation in geographic regions other then Northern/Central Europe. Such analyses would also allow to assess, if the rye collection assembled for this research is representative of the worldwide diversity.

Finally, a question might be posed to what extent the obtained results reflect the factual genetic relationships in the assembled rye collection. DArT markers are biallelic, which imposes certain limitations with the respect to the range of analyses that can be performed [29]. In addition, DArT markers are scored as dominant markers, in general, and it was done so also in this study. These features of DArT markers are of particular importance in the presented research, since most of the rye accessions analyzed are likely to be highly heterozygous and heterogeneous and to vary in allelic frequencies for a number of markers. Therefore it could be expected that the accuracy of the obtained results is compromised to some extent. The unquestionable advantage of DArT markers, however, that in our opinion counterbalances the potential drawbacks of assessing genetic variation in an outcrossing crop using this method, is the genome coverage achieved – in this study, more than one hundred DArT markers were available for each chromosome. Moreover, it was demonstrated by Laido *et al*. [27] that in tetraploid wheat the estimates of genetic diversity between genotypes obtained independently using SSR and DArT were highly similar and that dendrograms obtained with the two types of marker data were highly congruent. A comparison of SSR and DArT markers in assessing genetic diversity of 436 heterozygous cassava accessions also revealed that both markers systems generated similar clustering patterns [69], while in an empirical evaluation of DArT, SNP and SSR markers for genotyping, clustering and assigning sugar beet hybrid varieties into populations it was found that the same accuracy of the results can be achieved when 4.9-13.3 times more DArT markers then SSR markers are used [47]. Therefore, in reverse, it could be supposed, that our data is comparable with results from almost 80 SSR (1054 /13.3 = 79.25). By contrast, so far only much smaller numbers of SSR markers were used in genetic diversity analyses of rye: 24 [5], 30 [59], 38 [15], 10 [7]. Consistence of DArT based clustering with taxonomical classification was reported in a study of *Physaria* and *Paysonia* accessions [12] and in tetraploid wheats [27]. Our results obtained in cluster analysis of wild *Secale* germplasm also indicate that DArTs markers reflect phylogenetic relationships faithfully, even when applied to analyses of heterozygous and heterogenous samples, such as out-crossing rye accessions. Therefore we think that DArT based analyses provide a realistic picture of the genetic diversity and population structure present in the 379 rye accessions. Apart from high discriminatory power, faithful reflection of phylogenetic relationships, extensive genome coverage and the availability of information on chromosomal location, the use of DArT markers offers advantages also in terms of transferability, allowing for cross studies comparisons, and much lower costs compared to the other SNP genotyping platform currently available for rye – Illumina iSelect HD Custom BeadChip [70]. Apart from lower cost-effectiveness, diversity studies based on Illumina iSelect platform (especially studies involving highly diverged germplasm pools) are likely to be affected by ascertainment bias caused by the SNP discovery process in which limited number of individuals from selected populations are used. It was shown that this ascertainment bias can distort measures of population divergence [71]. Additionally, the ongoing sequencing of DArT markers from rye genotyping panel will soon enable more in depth analyses, such as candidate gene identification. DArTs can be thus recommended as a method of choice for rye germplasm characterization, as well as for association mapping studies, where the knowledge of population structure and genetic relationships is of critical importance [41,42].

## Conclusions

We conducted the largest and most comprehensive study of genetic diversity in rye to date and revealed a relatively limited diversity in improved rye accessions, both historical and contemporary, as well as lack of correlation between clustering of improved accessions and geographic origin, suggesting common genetic background of rye accessions from diverse geographic regions that probably results from extensive germplasm exchange. Genetic distinctness of contemporary varieties from the rest of accession that was also observed indicates a great genetic potential of *ex situ* collections for broadening the genetic diversity in current rye breeding programs.

One of the more interesting findings of our study is the identification of accession source as a major determinant of population structure. Our result point to possible influence of reproduction methods on the observed diversity pattern. Further studies involving accessions from different genebanks would be desirable to verify our findings concerning the source of population structure in rye.

We also revealed differences in distribution of genetic polymorphism among rye chromosomes that could be indicative of genome regions targeted by selection during domestication and improvement programs.

Obtained data indicate that DArT markers provide a realistic picture of the genetic diversity and population structure present in the collection of 379 rye accessions and are currently a method of choice for rye germplasm characterization and association mapping studies.

## Competing interests

AK is an employee of Diversity Arrays Technology Pty. Ltd., which offers genome profiling service using the technology described in this report. This fact, however, has not interfered whatsoever with the full, objective, transparent and unbiased presentation of the research results described in the manuscript nor alters the authors’ adherence to all the BMC policies on data and material release.

## Authors’ contributions

HBB conceived and designed the experiments, wrote the manuscript. HBB, MT performed the experiments. HBB, AK, LB analyzed the data. HBB, AK, LB, MT, MRT contributed reagents/materials/analysis tools. HBB, MT, MRT contributed to the selection and preparation of germplasm. All authors read and approved the final version of the manuscript.

## Supplementary Material

Additional file 1: Table S1 Excel spreadsheet containing detailed information on rye used in the study and their genotypic DArT marker data. Click here for file

Additional file 2: Table S2 Excel spreadsheet containing mean PIC values for groups of accessions created according to source and type of germplasm. Click here for file

Additional file 3: Figure S1 (Portable Document File). Delta K values for K values (number of populations assumed) ranging from 1 to 20. Click here for file

Additional file 4: Figure S2 (Portable Document File). Principal coordinate analysis of 379 rye accessions based on 1054 DArT markers with defined chromosomal location. Accessions were labeled according to the geographic origin of accessions: panel a: only cultivated materials and varieties from PAS BG are shown; panel b: only landraces are shown. Click here for file

Additional file 5: Table S3 Excel spreadsheet containing results of AMOVA and pairwise population Ph_PT_ analyses for accession source and improvement status, and for region of origin. Click here for file
