# Supplementary figures and images for: Genome-wide characterization of genetic diversity and population structure in Secale
Source: BMC Plant Biol. 2014 Aug 1;14:184. doi: 10.1186/1471-2229-14-184 (PMC4236688; doi:10.1186/1471-2229-14-184)

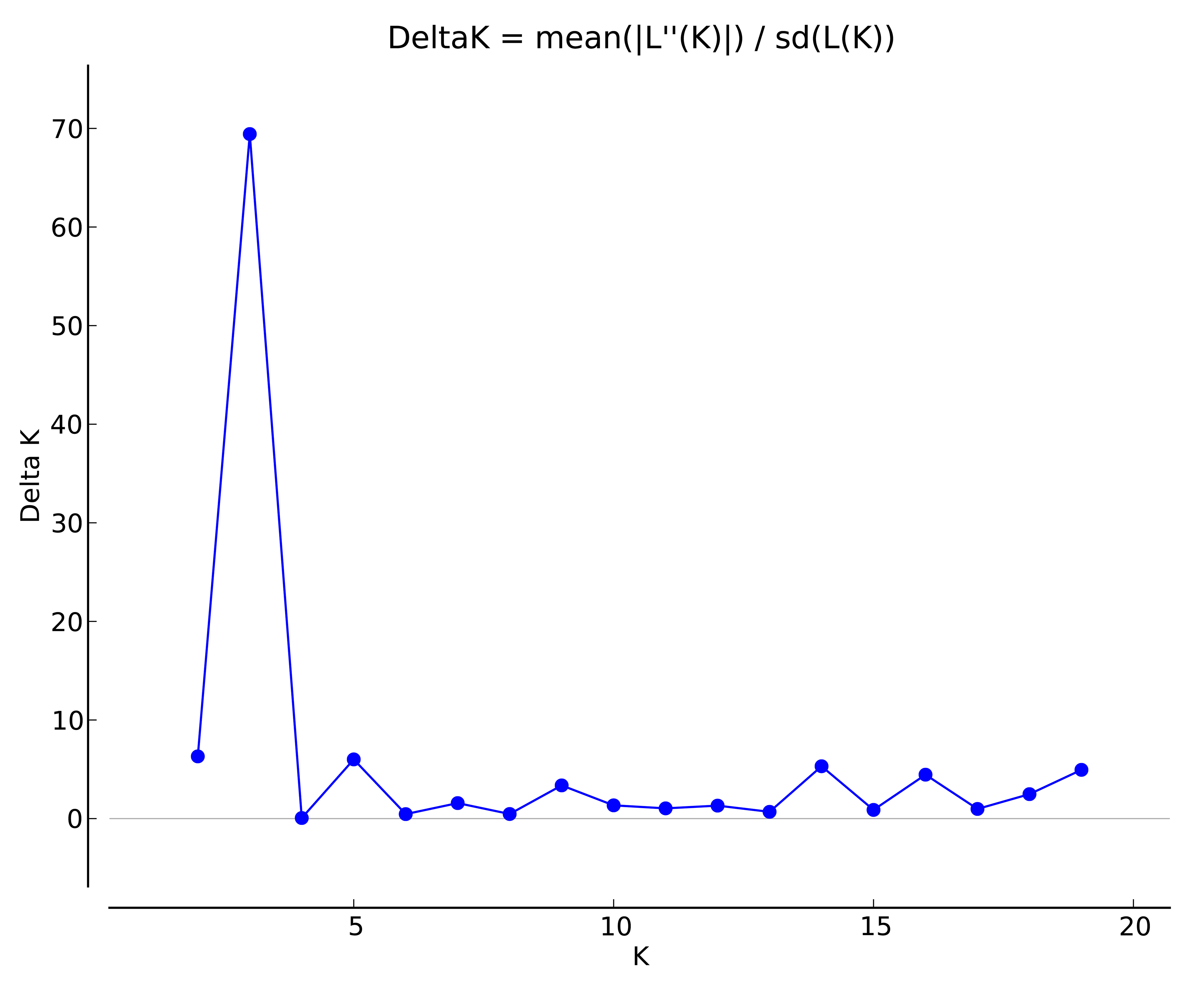

Supplement: Additional file 3: Figure S1 — (Portable Document File). Delta K values for K values (number of populations assumed) ranging from 1 to 20. [file 1471-2229-14-184-S3.tiff]

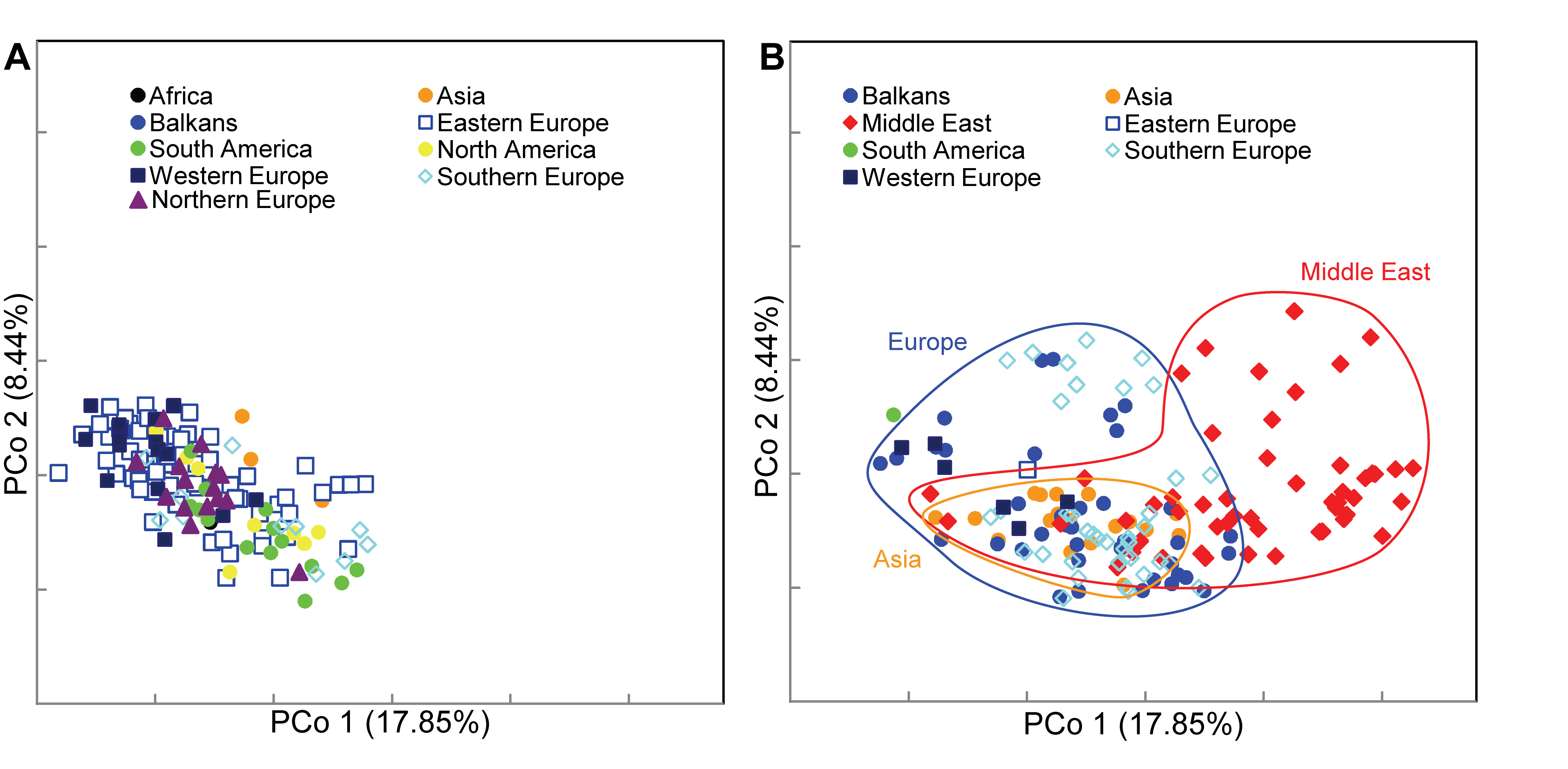

Supplement: Additional file 4: Figure S2 — (Portable Document File). Principal coordinate analysis of 379 rye accessions based on 1054 DArT markers with defined chromosomal location. Accessions were labeled according to the geographic origin of accessions: panel a: only cultivated materials and varieties from PAS BG are shown; panel b: only landraces are shown. [file 1471-2229-14-184-S4.tiff]
